# Cultural heritage training in the US military

**DOI:** 10.1186/2193-1801-3-126

**Published:** 2014-03-06

**Authors:** Leedjia Svec

**Affiliations:** Defense Equal Opportunity Management Institute (DEOMI), 366 Tuskegee Airmen Drive, Patrick Air Force Base, Brevard County, FL 32935 USA

**Keywords:** Culture, Military, Training, Heritage, Education, Preservation, Property, Government

## Abstract

Cultural competence is a vital component of many missions in today’s military. Cultural competence enables one to further a mission, save resources, and save lives. Conversely, a lack of cultural competence may bring about challenges to mission completion, requirement for more resources, waste of resources, and destruction of lives. Cultural competence involves many components. One particular component is cultural heritage awareness and protection of cultural property. This study sought to assess current understanding of cultural property protection and determine the effectiveness of a training aimed at increasing cultural property protection awareness, knowledge, and comfort within the military setting. It was hypothesized that participants would vary in their level of awareness, knowledge, and comfort of cultural property protection, and that all would show a significant improvement in knowledge scores post training. Factors such as deployment experience were examined for potential correlation with measures such as awareness. A 14 question pre-read survey was developed to assess participants’ demographics, awareness, knowledge, and comfort with cultural property protection. Awareness included value, laws, and procedures while knowledge examined “know how” such as how to bed down in a protected structure or communicate information about the structure. Comfort assessed one’s comfort with engaging in the knowledge based tasks. A 24 question post read survey was administered to assess awareness, knowledge, and comfort, and to solicit additional feedback on the manual itself. The survey utilized a 1–5 rating scale with 1 representing no awareness, knowledge, or comfort and 5 representing absolute awareness, knowledge, and comfort with different aspects of cultural property protection. Cultural property protection value was highest pre and post training while knowledge regarding recovery of property was rated lowest pre and post training. Results are encouraging for the pursuit of cultural property education. Further studies should include knowledge assessment versus self rating as well as tracking of incidents and outcomes in the field. Implications for mission readiness and success are discussed.

## Background

Cultural competence is a vital component of many missions in today’s military. Cultural competence enables one to further a mission, save resources, and save lives. A recent deployment of personnel in Libya took cultural competence into account enabling precise and efficient actions that saved local heritage and garnered support and respect of the local population (L. Rush, personal communication, October 17th, 2013). Conversely, a lack of cultural competence may bring about challenges to mission completion, requirement for more resources, waste of resources, and destruction of lives; in 2007 for example “$2 million was misspent” in relation to a historic citadel that was damaged during construction (Rush and Bogdanos 2009). As Colonel Mark Baines, Commandant of the NATO School in Oberammergau states, the danger of not having cultural competence is “mission failure, flat out” Baines (2012).

Cross Cultural Competence (3C) does not have a single definition. However, disciplines ranging from education and healthcare to business and military incorporate similar elements. The definition provided by Abbe et al. (2008) for example; “cross-cultural competence refers to the knowledge, affect/motivation, and skills that enable individuals to adapt effectively in cross-cultural environments” is built upon by Sands and Greene (2013) with the addition of the terms “abilities and attitudes” offering “a suite of competencies and enablers” that are crucial to 21st century military actions. As military members venture into new terrains, they must face other cultures on multiple levels. From shared language to engaging in proper societal customs, there is a vast array of well studied aspects of 3C. One aspect that may seem less obvious is cultural heritage and cultural property protection awareness. In gazing at the sculpture of *The David*, modern day philosopher Kwame Appiah remarks “I know that Michelangelo made a contribution to the culture of the world” (pg. 126). In all corners of the Earth, there are similar pieces of heritage, for example the Nok sculptures of Nigeria, belong to the entire world and “are of potential value to all human beings” (Appiah 2006 pg 120). As military members expand into other theatres of engagement, they will encounter sculptures, paintings, instruments and other items of cultural significance. How will they respond? Will they have culturally competent skills and attitudes? It is the belief of the author that one particular component of cultural competency is cultural heritage and the protection of cultural property, and that it is critical to future operations.

“Cultural heritage is defined as archaeological sites, sacred places, historic structures, and monuments” (Rush and Bogdanos 2009). Cultural property is comprised of the physical, social, and psychological components that define one’s culture. This may be a representation of a deity, a sacred space, a social practice such as going to the market, or a belief such as a local legend (Rush 2012a). Cultural heritage lays the foundation “for vibrant, innovative and prosperous knowledge societies” (UNESCO 2008). The cultures to which these items belong are the owners; disregarding this fact may lead to severed connections, poor communication, retaliation, poor public relations, and even violence (Matsuda 1998).

There are many news headlines featuring militaries behaving poorly toward others’ cultures, unintentionally or intentionally, actions that disregard cultural heritage may be harmful. In 2009, for example, U.S. forces expanded their camp in Afghanistan without taking the local culture and landscape into account (Philips 2009). As a result, ancient but still utilized water systems were blocked off or contaminated, upsetting the local villagers. The U.S. then had to pay reparations and was not able to work with the locals as intended. The impact of cultural heritage mistakes is significant and harmful; however there are also examples of military respect for cultural heritage. The recent coordination of the no-strike list heritage information between coalition forces enabled the U.S. and the U.K. to demonstrate respect for the cultural heritage of Italians and Libyans, for example (C. Wegener, personal communication, October 17th, 2013). Heritage preservation is a force multiplier and offers the opportunity to aid in rebuilding relations within countries. It is a way to show respect to coalition forces and generate valuable opportunities to partner in positive ways. It also contributes to unified operations, may save lives and dollars. Both the positive and negative illustrations point to the importance of cultural heritage education and training in the military.

What is the current state of understanding and training for military members with regard to cultural heritage? Numerous centers and resources exist for the purpose of cultural education. Sands and Greene (2013) note for example that “3C permeates DoD policy, doctrine, strategy and operations and is now being institutionalized in DoD military and civilian education and training”. While cultural competence is highly studied, a search of the literature reveals few studies with regard to cultural heritage specifically.

The extent of existing cultural heritage knowledge within the United States military is little known. Expanding cultural heritage preservation skills may be an untapped resource for allied forces. A series of studies was conducted to assess current understanding of cultural property protection within the U.S. military and to determine the effectiveness of a training aimed at increasing cultural property protection awareness, knowledge, and comfort within the military setting. It was hypothesized that participants would vary in their level of awareness, knowledge, and comfort of cultural property protection, and that all would show a significant improvement in knowledge scores post-training. Factors such as deployment experience were examined for potential correlation with measures such as awareness.

### Study one results

Study one utilized the full-length manual and participants primarily from DEOMI’s Leadership Team Awareness Seminar (LTAS). A total of 30 participants engaged in the study. All participants received the pre-test and post-test, 18 participants received the manual while 12 did not receive the manual for control. Participant demographics are illustrated in Table 1.

**Table 1:** **Study one demographics**

Gender	75% male and 25% female
Race	58% white, 33% black, and 9% other
Service	53% army, 13% navy, 13% marines, 9% air force, and 12% civilian
Rank	37% E-7–E-9, 37% O-4–O-6, 10% O-1–O-3, 10% GS-11–GS-14, and 6% other
Occupation	35% HR, 17% infantry, 17% science,14% legal, 10% supply, and 7% aviation
Deployed status	63% deployed and 37% not deployed
Cultural training	93% no cultural training and 7% cultural training
Witnessed destruction	Pre 8% witnessed destruction 92% no witness
	Post17% witnessed destruction, 83% no witness

Demographics and training status of participants in Study One.

**Figure 1:**
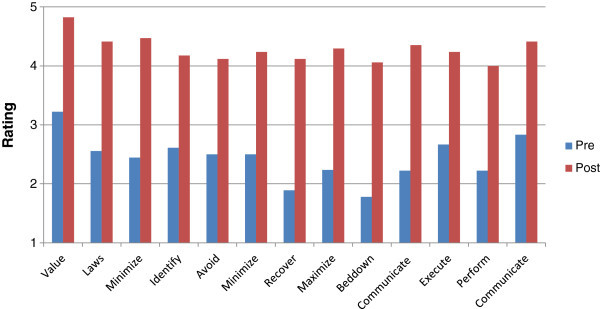
**Study one average ratings pre and post manual.** This figure illustrates the pre and post ratings of participants who received the manual for Study One. Value is rated highest both pre and post training with the manual. Bedding down with cultural property is rated lowest pre manual while performing CPP is rated lowest post manual.

Average pre-read scores indicated that participants had limited awareness for all measures regarding CPP (Figure 1).

**Figure 2:**
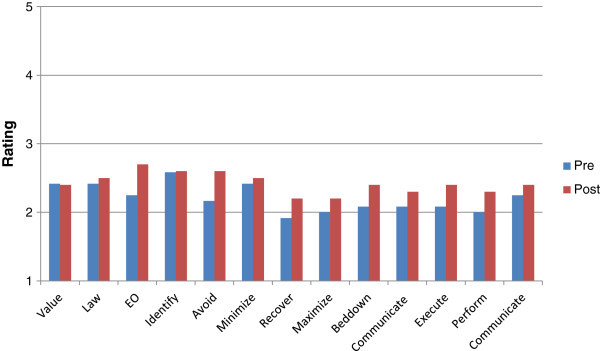
**Study one average ratings pre and post no manual.** This figure illustrates the pre and post ratings of participants who did not receive the manual for Study One. Participants scores do increase but do not vary significantly.

Participants averaged a statistically significant (P-value 0.0 level) 2-point increase in all measures on the post-test, indicating they were more aware, knowledgeable, and comfortable with CPP after reading the manual. Participants in the control group showed limited awareness for all measures regarding CPP (Figure 2) but did not show the increase in rating scores with post-assessment (P-values ranged from .24-.49), supporting the idea that the change in ratings was facilitated by the manual.

One hundred percent of participants agreed that the manual would be helpful for deployments and if they were given the manual they would read it. Participants rated the manual as extremely useful on average and found the formatting just right with elements (such as pictures or lists) in place.

### Study two results

Study Two utilized the shorter manual and participants from the Equal Opportunity Advisor Course (EOAC). A total of 79 participants engaged in the study. In the test group, 27 participants received the pre-test and post-test and the manual, while 52 participants served as control, engaging in pre-assessment surveys. Participant demographics are illustrated in Table 2.

**Table 2:** **Study two demographics**

Gender	58% male and 42% female
Race	51% black, 25% white, 10% hispanic, 10% other, and 4% Asian
Service	58% army, 22% air force, 11% navy, 3% marines, 3% coast guard, and 3% national guard
Rank	67% E-7–E-9, 17% E-4–E-6, 10% O-4–O-6, and 6% O-1–O-3
Occupation	42% HR, 14% supply, 11% other, 10% infantry, 8% EO, 6% medical, 3% signal, 3% aviation, and 3% legal
Deployed status	87% deployed and 13% not deployed

Demographics and training status of participants in Study Two.

**Figure 3:**
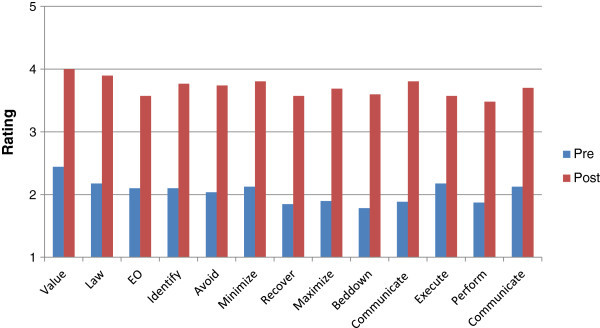
**Study two average ratings pre and post manual.** This figure illustrates the pre and post ratings of participants who received the manual for Study Two. Value is rated highest both pre and post training with the manual. Recovering and bedding down with cultural property is rated lowest pre manual while the connection to Equal Opportunity as well as performing CPP is rated lowest post manual.

Average pre-read scores indicated that test-group participants had limited awareness for all measures regarding CPP (Figure 3).

Participants averaged a statistically significant (P-value 0.0 level) 2-point increase in all but two measures which had a significant 1 point increase on the post-test, indicating they were more aware, knowledgeable, and comfortable with CPP after reading the manual. The two factors that had only one point significant increase in rating were awareness of the connection between cultural heritage and EO and comfort in execution of CPP.

**Figure 4:**
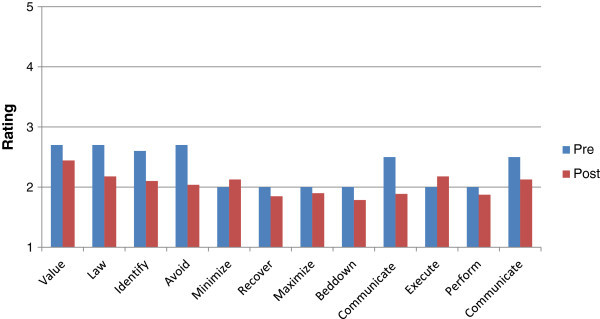
**Study two average ratings pre and post no manual.** This figure illustrates the pre and post ratings of participants who did not receive the manual for Study Two. Participants scores were higher in the pre no manual condition but do not vary significantly.

Participants in the control group showed limited awareness for all measures regarding CPP (Figure 4) but did not show the statistically significant increase in scores with post-assessment (P-values ranged from .24-.49), supporting the idea that the change in scores of the test group was facilitated by the manual.

The majority, but not all, of participants indicated that the manual would be helpful for deployments and that if they were given the manual, they would read it. Participants rated the manual as useful on average and found the formatting just right with elements (such as pictures or lists) in place.

### Study three results

A total of 106 participants judged scenarios that involved hazing or bullying actions toward others’ property. Total average ratings revealed that the majority of participants (50%) were not sure how to view the destruction of other’s cultural property 45% viewed the scenarios as hazing or bullying while 5% of participants viewed destroying other’s cultural property as “just having fun”.

## Discussion

Participant demographics were not equal in category representation, with both studies having a majority of Army, male, human resources (HR), deployed, and persons from the senior enlisted ranks who had not received cultural heritage training nor witnessed cultural heritage destruction. While comparisons could be made on the bases of job, service, gender, etc., they would be limited due to the unequal sample size. Additionally, no Clandestine Services were surveyed. Future efforts may benefit from obtaining their input, particularly Clandestine Services who are frequently in forward deployed environments.

Pre-test read the majority of participants for both studies had limited awareness of cultural heritage laws and minimization of damage. These findings are interesting in that a significant number of military members deploy to foreign areas where the awareness of cultural heritage can save lives and dollars. Additionally, a sizable number of participants remarked that this was novel and important information. The value of cultural heritage stood out from laws and minimization as participants had the highest ratings of this measure pre- and post-test for both groups. It follows that the value of one’s cultural heritage may be more easily grasped; however, it is possible that the relevance to the military may need further strengthening for some participants.

Examination of knowledge scores pre-test read shows that variations exist within cultural knowledge domains, highlighting certain domains as being less known among the participants tested. The majority of participants in both groups did not know how to recover or how to beddown with cultural property, for example. Post-test read these scores had significantly improved but still remained the lowest scores. This finding is important in that cultural property is purposely utilized by enemy forces and can be a source of protection from enemy fire. It follows that knowledge about bedding down with cultural property would be one of the first domains in which to target training. It is also likely that more interactive training is required for these domains as opposed to the written format utilized in the manual. On average, however, participants’ knowledge significantly increased for all knowledge measures after reading the manual for both groups.

**Figure 5:**
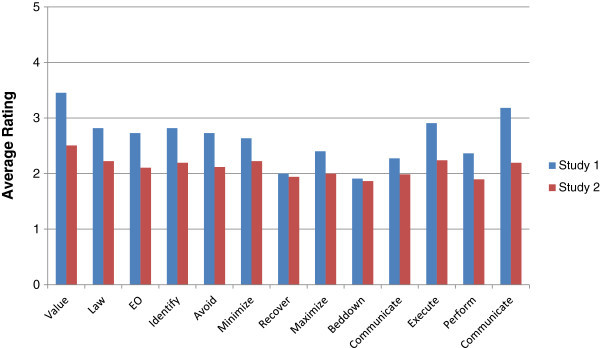
**Comparison of deployed participants pre training.** This figure illustrates the pre manual ratings of participants who deployed from both studies. Participants in Study One exhibit higher ratings than those of Study Two for all measures. Value has the highest rating for both groups; however participants from Study One are much higher. Recovery and bedding down with CPP have the lowest ratings for both groups.

It is worth noting that the groups did not start out with the same scores on average, with the senior leaders having higher pre-read averages than the non-senior leaders, which would be expected. The cause of this difference could be due to seniority and confidence or experience. While it is not certain which factor contributed more, deployment levels were higher in the second group, reducing the likelihood that deployment experience led to greater knowledge between the two groups. This factor was isolated and examined. The pre-manual ratings of those who had deployed for Study One and Study Two were compared via independent t-tests revealing Study 1 participants having greater average ratings for all factors (Figure 5).

Differences were statistically insignificant (P-values ranged from .12 to .86) between all but two factors, awareness of the value of CPP and comfort with communication regarding CPP, which were statistically significant (P-value .04 and .03, respectively). The difference in ratings appears to be more likely due to differences in the seniority of the first group rather than deployment status. While the number of participants within each rank was too small for a proper statistical test, a visual comparison of the data between deployed groups broken down by rank revealed that those with more seniority tended to have higher ratings across the board in both studies.

Many studies in other fields, such as HIV prevention, have shown that knowledge does not equal efficacy or a sense of comfort or belief that one is capable of changing behavior despite knowledge that behavior should be changed (Svec and Wang 2003). Comfort with cultural heritage was assessed to gauge whether participants had the confidence to engage in cultural property protection behaviors after learning how to do so. The majority of participants were not comfortable with performance pre-test read; however, a sizable number of participants were comfortable despite not being fully informed. Comfort with communication was higher, while comfort with execution was lower. It is encouraging that knowledge increased efficacy and interesting that participants could be confident in skills they did not have. This finding highlights the need for objective data that assess cultural competence and heritage preservation skills as well as subjective data. People who are confident but inaccurate may do more damage with regard to cultural heritage preservation (or any skill).

**Figure 6:**
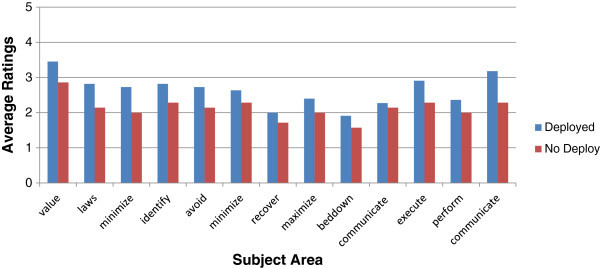
**Study one average pre manual ratings by deployment status.** This figure illustrates the ratings sorted by deployment status (deployed or not deployed) of participants from Study One. Those who had not deployed showed lower ratings for all measures.

**Figure 7:**
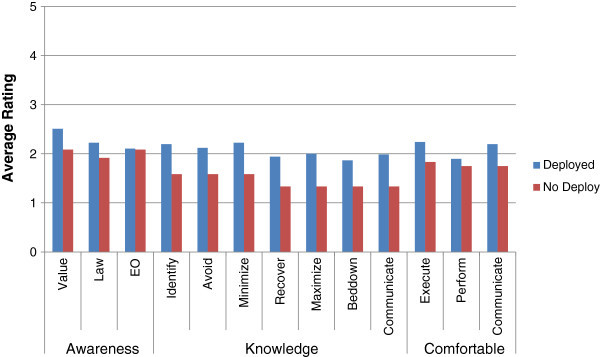
**Study two average pre manual ratings by deployment status.** This figure illustrates the ratings sorted by deployment status (deployed or not deployed) of participants from Study Two. Those who had not deployed showed lower ratings for all measures.

One consideration when examining the data is that deployment status had an influence on levels of CPP awareness, knowledge, and comfort pre training. To examine whether this was the case, participants’ data were separated into either having been deployed or never deployed and compared. As would be expected, those who had deployed rated all measures higher (greater awareness, knowledge, comfort) than those who had not deployed, pre-test read, for all measures for both studies (Figures 6 and 7).

The difference, was not significant for Study 1 (Study 1 P-values ranged from .19 to .80), while Study 2 showed statistical significance in all of the knowledge factors (by 1 rating on average) but none of the awareness or comfort factors (P-values were .03, .04, .02, .01, .00, .01 and .00 for knowledge factors of identifying, avoiding damage, minimizing damage, recovering, maximizing, bedding down with, and communicating CPP, respectively. Non significant P- values ranged from .15 to .48 for the other factors. While deployment does increase all CPP self-ratings, it is likely that deployment in itself is not sufficient to provide all the necessary skills one needs to be culturally competent. Factors such as awareness and comfort may be less subject to experience while knowledge and skills are aided by the experience of deployment. It would be curious to test the pre cultural heritage training ratings of those who have not deployed but have gone through realistic deployment training, such as found at Fort Drum, NY. It follows that higher ratings could make for higher proficiency before one is tested by the reality of a cultural heritage in combat situations; however, this remains to be tested objectively.

The scenario assessment data in Study Three was illustrative in that a large percentage of participants were unsure how to view cultural heritage situations and that there are participants who would purposely destroy other’s heritage for fun. When considering the knowledge and abilities component of 3C with regard to cultural heritage and property protection, it is clear that there is room for improvement. It is not clear however, if the source of uncertainty or a desire to destroy other’s heritage is a function of job type, attitude, or lack of knowledge. Future studies would benefit from including these types of questions in the pre-post assessment to determine whether education would shift participants’ scenario judgments. It is possible that training would not modify such beliefs however; several comments reflected a belief that destroying other’s culture was fun and/or necessary for survival. Future studies would benefit from including questions of attitude and correlation analysis. While these beliefs appear to be present, are concerning, and must not be ignored, it is encouraging that they are reflected in a small percentage of participants.

Assessment of the manuals themselves revealed that participants found them usable. The lowest rating, that for pictures, was likely due to image quality as participants remarked that they were hard to decipher. The manual was printed in black and white; therefore, it is important to ensure color printing of the photographs for maximum effectiveness before the manual is distributed. The manual content was well received. However, the length of the manual was rated as a little too long, which was expressed in the comments. One of the purposes of the manual is use in the field; therefore, a short and direct version via app, e-reader, or pocket device may be worth pursuing in conjunction with publication of this manual for further reference.

Suggestions included checklists at the end of each section, increasing and clarifying the “so what” factor, and electronic or PowerPoint formats. Service members may appreciate a version that could be viewed on their e-readers. The most frequent comments included the importance of cultural heritage knowledge, the novelty of this knowledge to the participants, and the need for more segments of the military to have this knowledge.

It is worthwhile to pursue versions that may be aimed at different segments of the population, from the senior leaders to the newly enlisted, as well as service specific cultural property knowledge. What Air Force pilot encounters may be vastly different from what a Navy diver deals with; however, both situations are important. Cultural heritage is a legal matter as well as a human rights, EO, and cultural competence matter. The more often troops are socialized to these concepts, the less often there should be international incidents of cultural heritage actions gone wrong.

## Conclusion

Cultural property and its protection is a matter of law, heritage, human rights, and strategy. Few formal studies have been conducted with regard to CPP and the military; training CPP as well as understanding the current state of it in the military remain an important and needed area for understanding. Such information can be utilized to guide policy, training, and future directions. This study sought to assess the current state of cultural heritage awareness, knowledge, and comfort as well as the effectiveness and areas for improvement of the cultural heritage training manual.

The results of this study indicate that the current state of cultural heritage awareness and knowledge among service members has room for improvement. Participants somewhat know the value of cultural heritage and are less than somewhat aware of laws or protection. This finding is important because service members will still be held accountable to the law, even if they do not know it. With regard to cultural heritage knowledge, participants are not really knowledgeable; however, this varied with deployment. Participants were somewhat comfortable with cultural heritage, and those with more knowledge were more comfortable, as one would expect. While further studies would be required, it appears that the knowledge provided was enough to increase efficacy in engaging cultural heritage for the vast majority of participants. Despite variations between participants’ base knowledge, participants’ average scores clearly increased in all three areas after reading the training manual.

Cultural property protection value was highest pre- and post-training, while knowledge regarding recovery of property was rated lowest pre- and post-training. Differences between those who had deployed were minimized post training (no significant differences were found). This finding is important in that while not all participants began with the same levels of awareness or knowledge with cultural heritage, they finished relatively the same. This is encouraging for maintaining an equal playing field and equitable resources and capacities among service members as they engage in cultural heritage practice and preservation.

Future studies should include objective assessment of awareness and knowledge rather than, or in addition to, self rating. Additional questions should also assess training and experience with cultural heritage specifically. It is known, for example, that the Army has Fort Drum as a place to engage in cultural heritage education and that certain career fields, such as law have courses in cultural property; however, it is not known whether other branches have such resources. An additional measure would be a follow up study to ensure retention of knowledge after training, as well as re-test reliability. Lastly, tracking of incidents and outcomes in the field may be the key to policy and leadership support and ownership. While these factors are known, objective measurement and illustration would be a significant next step.

With regard to the manual itself, revisions to create a slightly shorter interactive, dynamic, electronic version is recommended. Different educational levels or purposes could be embedded in different leadership levels within the military. The recommendation for collateral duty is also worth consideration. Just as participants receive an in-depth training that allows them to help others vote, be fit, volunteer, keep track of hazardous substances in medical clinics, and more, commands or units that would benefit from cultural heritage training could employ this as a collateral duty. Training could occur at Fort Drum as well as online. This would enable a streamlined advocacy and reach-back capability that service members remarked they needed.

These studies sought to examine the following questions: Do service members have the necessary skills to protect cultural property as they deploy worldwide? Do service members see the impact of cultural property protection on matters that range from equal opportunity to national security? What is the impact of cultural property training? While the current studies leave room for further refinement and methodological improvement, they do lend data that is helpful to exploring these questions.

The vast majority of participants had no experience with cultural heritage training, and had some awareness of cultural heritage value but little cultural heritage knowledge or efficacy. Participants varied in their understanding of the connection between cultural heritage and EO or national security as evidenced by specific questions and analysis of their comments. Several participants, for example, believed that cultural heritage did not apply to them, while after the training, several participants viewed destruction of cultural property in new light. The impact of cultural property training was measurably significant, with the majority of participants improving on all measures. The biggest difference between the two manuals, as shown in the data, was in assessment; 100% of participants found the longer manual useful and would read it, while this was not the case with the shorter manual. Further studies would be required to determine whether this finding is a function of group difference or manual difference.

In conclusion, protection and preservation of cultural heritage is an important process and outcome. Cultural heritage relates to issues faced in deployment as well as in times of peace, in land and on sea. The sheer volume of participants who have deployed but have not received cultural heritage information is alarming; however, the effectiveness of training is encouraging. In conclusion, the concept of cultural heritage is moderately known in the field, has the ability to be successfully taught, and remains an important component of today's forces' intellectual toolkit. As Bokova notes, the importance of cultural heritage cannot be overstated; it is “a driver and enabler of sustainability … a source of meaning and belonging … a wellspring of creativity and innovation essential for all societies today” Bokova (2013).

## Methods

A 14-question pre-read survey was developed to assess participants’ demographics, awareness, knowledge, and efficacy with regard to Cultural Property Protection (CPP). Demographics included questions on CPP training and cultural property destruction. Awareness included values, laws, and procedures, while knowledge examined know-how, such as how to beddown in a protected structure or communicate information about the structure. Efficacy assessed one’s comfort with engaging in the knowledge-based tasks. After participants completed the pre-survey, they were either asked to read one of two hard copy manuals on CPP that they were given or they were given instruction in equal opportunity (EO) subjects. This study was approved by DEOMI Institutional Review Board and conducted in accordance with human subjects protection laws (IRB approval number CDO-14-6003).

The CPP manuals were developed by the Combatant Command (COCOM) Cultural Heritage Action Group (http://cchag.org/). Both manuals connected concepts of cultural property protection with well-established military operations concepts. One manual, “The Cultural Minefield: A Manual on Cultural Property Protection for the Operator Forward” (Rush 2012b) was 76 pages and took approximately 2 hours to complete. The other manual, “A manual for cultural property protection in the deployed environment” (Rush 2012c) was 12 pages and took no more than 45 minutes to complete. After reading the manual (or receiving general EO knowledge), participants completed a post-read survey.

A 24-question post-read survey was administered to assess awareness, knowledge, and comfort, in addition to feedback on the manual itself. The surveys utilized a 1–5 rating scale with 1 representing no awareness, knowledge, or comfort and 5 representing absolute awareness, knowledge, and comfort with different aspects of cultural property protection. Participants were solicited primarily in person. Participants were informed that it was a volunteer opportunity and that, should they decide to participate, they would fill out a pre-read survey, read the manual (or not), and complete a post-read survey.

Participants were divided into “Study One” and “Study Two” to reflect that they were recruited from different sources and received different manuals. Paired t-tests were conducted within each study using Excel database software.

The same participants who also engaged in Study Two engaged in a separate study that examined hazing. Participants were given scenarios of initiation, celebration, and bullying behaviors and were asked to judge what the behavior was for each scenario. Seven questions specifically examined judgment toward damage of cultural property. These questions were pulled from this study and are included for consideration and referred to as “Study Three”. Results were examined from the standpoint of descriptive statistics.

## Authors’ information

LS is a Lieutenant Commander in the United States Navy currently stationed at the Defense Equal Opportunity Management Institute as a senior policy, basic, and applied scientist, is a member of the Combatant Command Cultural Heritage Action Group and is an Annual Student Fellow of the Institute for the Study of Culture and Language at Norwich University.

## Abbreviations

COCOM: Combatant command
CCHAG: Combatant command cultural heritage action group
CPP: Cultural property protection
DEOMI: Defense Equal Opportunity Management Institute
DoD: Department of Defense
E: Enlisted
EO: Equal opportunity
EOAC: Equal opportunity advisor course
E-reader: Electronic reader
HR: Human resources
LTAS: Leadership team awareness seminar
O: Officer
p-value: Probability value
US: United States
UK: United Kingdom
3C: Cross cultural competence.
